# A Large Cohort Study on the Clinical Value of Simultaneous Amplification and Testing for the Diagnosis of Pulmonary Tuberculosis

**DOI:** 10.1097/MD.0000000000002597

**Published:** 2016-01-29

**Authors:** Liping Yan, Shenjie Tang, Yan Yang, Xiang Shi, Yanping Ge, Wenwen Sun, Yidian Liu, Xiaohui Hao, Xuwei Gui, Hongyun Yin, Ya He, Qing Zhang

**Affiliations:** From the Tuberculosis Clinic and Research Center, Shanghai Key Lab of Tuberculosis, Shanghai Pulmonary Hospital, Tongji University School of Medicine, Shanghai (LY, YY, XS, YG, WS, YL, XH, XG, HY, YH, QZ); and Tuberculosis Multi-Disciplinary Diagnosis and Treatment Center, Beijing Chest Hospital, Capital Medical University, Beijing, China (ST).

## Abstract

The AmpSure simultaneous amplification and testing method for the detection of *Mycobacterium tuberculosis* (SAT-TB assay) was designed to diagnose rapidly pulmonary tuberculosis (PTB). Unfortunately, the diagnostic advantage is unclear from previous small sample studies. In the current inquiry, a large sample size was used to reevaluate the clinical accuracy of the SAT-TB assay using sputum specimens.

A total of 3608 patients with suspected PTB were enrolled prospectively for diagnosis from sputum specimens using the SAT-TB assay. Of these, 2457 had a definite diagnosis of PTB confirmed by positive microbiology, or pathologic findings of TB in the lung, or clinical diagnosis of active PTB following anti-TB treatment with a favorable response. The sensitivity, specificity and accuracy of the SAT-TB assay were 75.8%, 100%, and 80.2%, respectively. The sensitivity of SAT-TB was significantly higher than that of the sputum smear (23.8%) (X^2^ = 1327.437; *P* = 0.000), wheresa significantly lower than that of sputum culture (89.0%) (X^2^ = 148.197; *P* = 0.000). The specificity of SAT-TB was significantly higher than that of sputum smears (96.3%) (X^2^ = 20.375, *P* = 0.000), whereas no significant difference was found compared with sputum cultures (99.6%) (X^2^ = 2.004, *P* = 0.500).

Positive results in the SAT-TB assay using sputum specimens indicates that active PTB is present and anti-TB treatment is strongly recommended regardless of smear and culture test results. Simultaneous amplification and testing method for detection of Mycobacterium tuberculosis is an accurate, cheap, and rapid method for PTB diagnosis.

## INTRODUCTION

Tuberculosis (TB) is a major public health problem worldwide. According to data from the World Health Organization (WHO), approximately 9 million people were newly diagnosed with active TB in 2013 and 1.5 million people died from the disease.^1^ Despite progress being made, rapid diagnosis, particularly of pulmonary tuberculosis (PTB), is still an obstacle.^2^ China is one of the 22 high TB burden countries in the world, with 1.5 million people infected with *Mycobacteria tuberculosis* (MTB).^3^ Although the prevalence of TB is gradually decreasing,^4^ it still remains an important threat because of its high infectiousness. The Chinese government is making strenuous efforts to provide rapid diagnosis and treatment facilities through national support and project grants. At present, the diagnosis of pulmonary TB is routinely made by bacteriologic verification from sputum or bronchial alveolar lavage fluid specimens.^5^ The acid-fast bacilli (AFB) smear test is the most commonly used method and offers rapid results, but has the disadvantage of poor sensitivity and does not discriminate TB from nontuberculous mycobacterial (NTM) infections.^6^ Mycobacterium culture is the gold standard for confirming TB but can take up to 6 to 8 weeks to obtain the results, rendering this diagnostic method inconvenient in routine clinical practice. Therefore, diagnostic capacity remains limited, especially in TB endemic countries like China but the development and adoption of new diagnostic tools will help to accelerate the diagnosis of TB.

The AmpSure simultaneous amplification and testing method for detection of Mycobacterium tuberculosis complex by using isothermal RNA amplification and real-time fluorescence detection has recently been introduced into a small number of Chinese hospitals for the early diagnosis of TB. It combines the technologies of nucleic acid isolation, simultaneous amplification, and testing with fluorescence-labeled hybridization probes. It is faster than conventional bacteriological methods and, importantly, it has excellent reproducibility.^7^ One previous study of SAT-TB assay focused on its accuracy with limited samples and demonstrated that its overall sensitivity for the diagnosis of PTB was 67.7%.^8^ Sensitivities for smear negative specimens were reported to be 39.2% and 93%.^8,9^ Although these studies were all carried out under controlled, standardized conditions, the results showed marked differences making it necessary to reevaluate the SAT-TB assay sensitivity with many more samples.

Thus, the current large sample study using sputum from suspected active PTB and HIV-negative adult patients was undertaken to assess the sensitivity, specificity, and accuracy of SAT-TB assays in real life situations in high TB burdened regions of China.

## PATIENTS AND METHODS

### Patients

The Ethics Committee of Shanghai Pulmonary Hospital approved this prospective study and written informed consent was obtained from each participant before enrollment. All adult patients admitted to the Shanghai Pulmonary Hospital were screened between January 2014 and April 2015. Inclusion criteria were: suspected active PTB; adults (≥17 years); no previous history of anti-TB treatment; negative HIV status. Exclusion criteria were: inability to provide sputum for examinations; no finalized diagnosis after examination and treatment (obscure diagnosis). A standard questionnaire was completed by each patient before enrollment, including basic demographic data, history of TB contacts, previous TB, current TB symptoms, anti-TB treatments as well as underlying diseases and concurrent therapies. All patients who were suspected of having active TB were tested using the SAT-TB assay, acid-fast bacilli AFB smear, and culture tests at enrollment, in addition to the T-SPOT. Interferon-gamma release assay as well as physical, pathologic, and radiographic examinations.

Active PTB was confirmed when radiographic or chest computed tomography (CT) manifestations were in accordance with PTB pattern and accompanied by one of the following criteria at enrollment or later during the study period:

- Bacteriologic diagnosis based on positive MTB in cultures
- Pathologic diagnosis of PTB based on analyses of resected lung tissues

If the patients did not meet one of the above 2 criteria, clinical diagnosis was confirmed by meeting all of the following categories: Patients with signs or symptoms of PTB and typical manifestation of TB on chest computed tomography; Patients received anti-TB medication for 2 months with a favorable response, based on improved signs or symptoms and chest CT results. For these cases chest CT was reviewed every 2 months until the patients were treated for 6 months. The clinical experts decided the final diagnosis of PTB, and they were blinded to the results of the SAT-TB assays; no evidence of non-TB lung disease based on laboratory and radiographic data; positive T-SPOT. Interferon-gamma release assays.

We excluded patients who had failed to meet the above 4 categories (obscure diagnosis). An evidence-based strategy was used to ensure that individuals with PTB were correctly diagnosed and had access to appropriate treatment as soon as possible, and also to ensure the quality of every step in the diagnostic process.

### Study Procedure

Clinical information, including treatment processes and the discharge diagnosis, were recorded from patients’ medical charts by investigators. For patients whose diagnosis was not established during hospitalization, an outpatient interview was conducted once a month after discharge to obtain the final diagnosis. At the end of the monthly follow-ups for 6 months, each patient was classified into one of 3 predefined clinical categories, namely active PTB, non-TB pulmonary disease or without a final diagnosis.

### Microbiological Identification

#### Acid-fast Bacilli Smears and Culture Assays

Morning sputum specimens were obtained before treatment. Sputum samples were routinely tested by smear fluorescence microscopy and by culture strain identification Lowenstein–Jensen medium, as well as the Bactec MGIT 960 System (Becton Dickinson Diagnostic Systems, Sparks, MD) according to World Health Organization guidelines.^10^ The detection threshold of a positive sputum smear was smear positive grade 1. All tests were performed at the tuberculosis reference laboratory in Shanghai Pulmonary Hospital, and quality control was routinely performed.

#### Simultaneous Amplification and Testing Method for Detection of Mycobacterium tuberculosis

Simultaneous amplification and testing method for detection of Mycobacterium tuberculosis was performed on sputum samples according to the manufacturer's instructions (Shanghai Rendu Biotechnology Co, Ltd). Sputum specimens were collected and transported to the laboratory, and then processed after being dissolved in sodium hydroxide. During the SAT- TB assay, the target 16S rRNA from MTB combined with T7 promoter fused primers was reverse transcribed into cDNA by Moloney murine leukemia virus reverse transcriptase. Then, multiple RNA copies were generated from the cDNA copy using T7 RNA polymerase, which were again transcribed into the T7 promoter, including cDNAs. The accumulated cDNAs were detected by fluorescence-labeled specific probes.

### Statistical Analysis

Statistical analysis was conducted using SPSS ver. 18.0 software (SPSS Inc, Chicago, IL). Assuming a positive rate of 93% for the SAT-TB assay in bacteriologically and clinically confirmed PTB patients,^9^ a sample size of 100 patients was required to conduct a power test at the 95% level. Sensitivity, specificity, positive and negative predictive values, as well as accuracy rates of the SAT-TB assay, sputum smear and cultures were calculated. Numerical variables are shown as the mean ± standard deviation. The categorical variables were analyzed using Fisher exact or Pearson χ^2^ tests where appropriate and 2-tailed tests were used. The concordance of agreement between SAT-TB assays and smear and culture data was assessed using Cohen kappa test (κ > 0.75, excellent agreement; 0.4 < κ < 0.75, moderate agreement; and κ < 0.4, poor agreement). *P* values ≤0.05 were considered to be statistically significant.

## RESULTS

### Study Patients and Results of Diagnostic Methods

A total of 3608 hospitalized patients with suspected active PTB were prospectively enrolled into the study.

**FIGURE 1:**
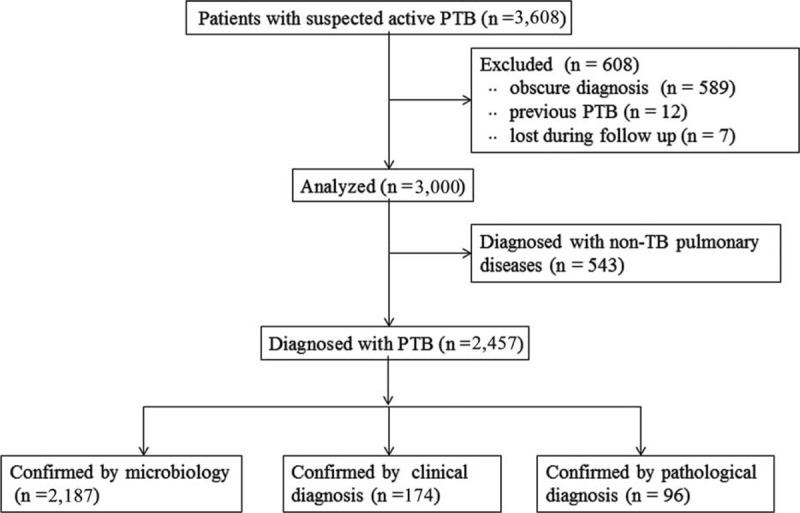
Flow chart of the study.

A total of 589 patients were excluded because of obscure diagnosis, 12 patients were excluded because of previously diagnosed PTB and 7 patients were excluded because of lost to follow up. When patients with obscure diagnosis had been treated for 2 months with anti-TB agents without improved chest CT results, the anti-TB agent applications were stopped. In these 589 patients, no individual was SAT-TB positive and no sputum smear AFB and mycobacterial culture test was positive. Finally, the data from 3000 patients were analyzed. According to comprehensive evaluations and follow-ups, 2457 patients (81.9%) were diagnosed with PTB and 543 patients (18.1%) with non-TB pulmonary disease. Among the 2457 active PTB patients, 2187 (89.0%) were confirmed by microbiology, 174 subjects (7.1%) by clinical diagnosis, and 96 subjects (3.9%) by pathologic diagnosis (Figure 1). In Table 1, the positive rates of detecting TB and non-TB pulmonary diseases are listed.

**TABLE 1:**
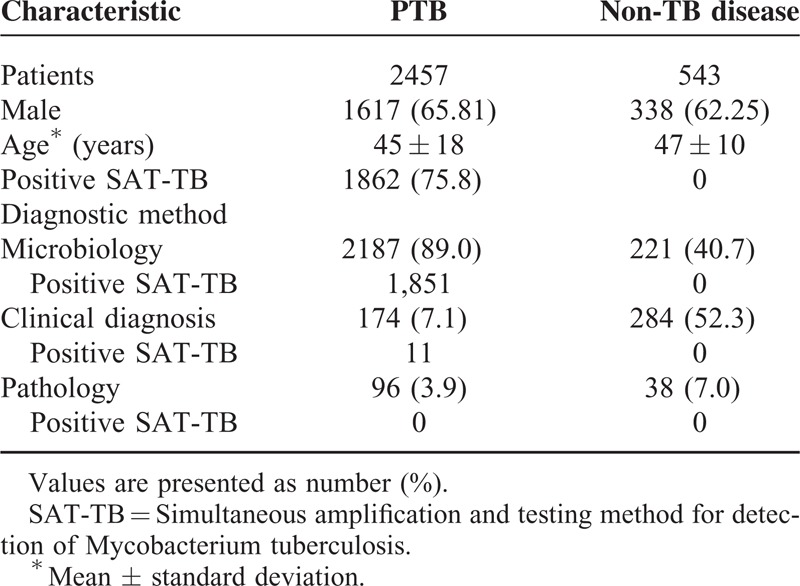
Overall Results of Different Diagnostic Tuberculosis Evaluations

### Performance of 3 Microbiological Sputum Assays

The sensitivity of SAT-TB (75.8%) for the diagnosis of PTB was significantly higher than that of sputum smears (23.8%) (X^2^ = 1327.437; *P* = 0.000), whereas significantly lower than that of sputum cultures (89.0%) (X^2^ = 148.197; *P* = 0.000). The specificity of SAT-TB (100%) for the diagnosis of PTB was significantly higher than that of sputum smears (96.3%) (X^2^ = 20.375; *P* = 0.000), whereas there was no significant difference with sputum cultures (99.6%) (X^2^ = 2.004; *P* = 0.5) (Tables 2 and 3).

**TABLE 2:**
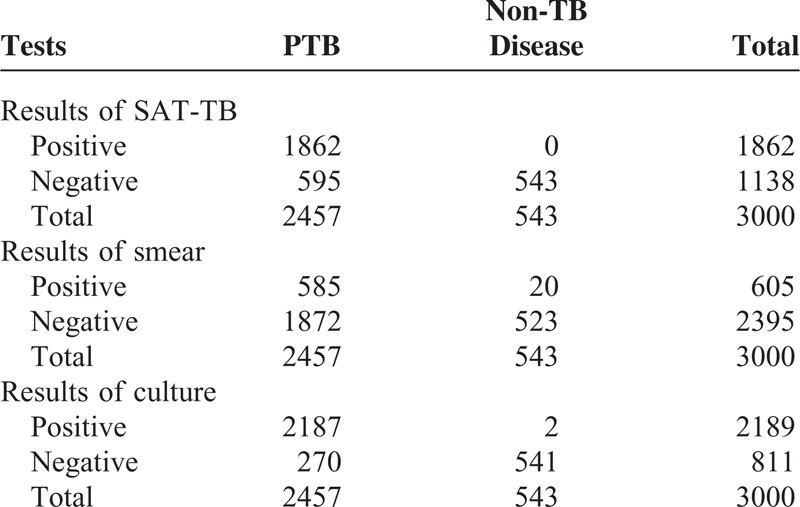
Results of 3 Tests for Detecting Tuberculosis in Sputum Samples

**TABLE 3:**
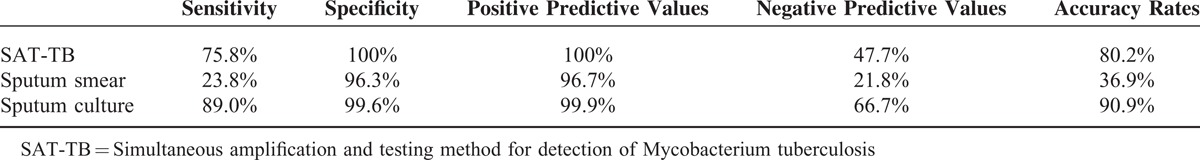
Comparison of Simultaneous Amplification and Testing Method for Detection of Mycobacterium tuberculosis, Sputum Smear, and Culture Results

For the 585 smear-positive PTB patients, the sensitivity of the SAT-TB assay was 86.8% and was significantly lower than that of sputum cultures (93.3%, X^2^ = 19.426, *P* = 0.000). Of the 1872 smear-negative PTB patients, the sensitivity of the SAT-TB assay was 72.3%, which was significantly lower than that of sputum cultures (87.3%, X^2^ = 131.001; *P* = 0.000).

### Combination of Simultaneous Amplification and Testing Method for Detection of Mycobacterium tuberculosis, Smear, and Culture Tests

Among the 2220 PTB patients who had positive sputum smears or cultures, 1851 also had positive SAT-TB results (1851/2220; 83.4%). In the 552 subjects whose sputum smears and cultures were both positive, the SAT-TB positivity rate was 91.5% (505/552) and in the entire culture positive group, SAT-TB detected 1848 of 2187 patients (84.5%). In the 33 solely smear positive cases, only 3 (9.1%) were also deemed positive with the SAT-TB test. Notably, SAT-TB detected 11 PTB cases, which were negative for both sputum smears and culture tests (Table 4).

**TABLE 4:**
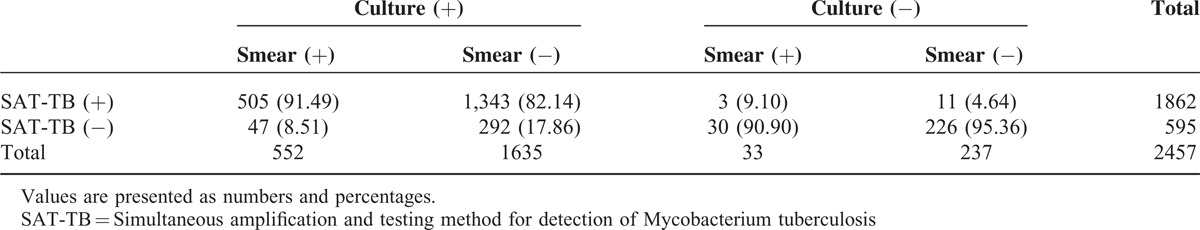
Combined Simultaneous Amplification and Testing Method for Detection of Mycobacterium tuberculosis Assay, Smear, and Culture Test Results

### Consistency Between Simultaneous Amplification and Testing Method for Detection of Mycobacterium tuberculosis and Sputum Culture

A moderate level of agreement (*n* = 3000; 88.2%) was found between the SAT-TB assay and sputum cultures (κ = 0.734, *P* = 0.000), indicating suboptimal agreement between the 2 tests.

## DISCUSSION

In our prospective study, we evaluated the clinical utility of sputum SAT-TB assays for the diagnosis of active PTB in TB endemic settings. The SAT-TB assay is preferred for the respiratory specimen, especially sputum samples, when PTB is suspected in clinical practice.

Many studies have demonstrated that PCR tests are routinely used for molecular diagnostic, for its rapidity and relatively high sensitivity and specificity, compared with conventional smear or culture tests. False-positive and false-negative results, however, are unavoidable with the current technology.^11–13^ The GeneXpert (Cepheid, Sunnyvale, CA) assay was introduced recently in China, but the high cost makes it unaffordable as a routine test,^14^ because the price is $100 in Shanghai public markets and not covered by the public medical insurance, whereas the cost for a SAT-TB assay is $30 and under medical reimbursement coverage. The SAT-TB assay provides another unique advantage over PCR, as the detection target is rRNA. RNA is much more labile outside the reaction tube than DNA amplification products. Because PCR detection commonly experiences contamination problems, the SAT-TB assay can reduce the risk of laboratory contamination and false-positive results. The sensitivity and negative predictive value of SAT-TB assay were lower than that of other TB RNA PCR tests available on the market (MTD test, Gen-Probe; San Diego, CA, USA).^15^ The MTD test, however, requires expensive specialized detection equipment, which prevents the assay from being widely adopted in resource-limited settings. The SAT-TB assay can be performed on real-time PCR instruments, which can be found in most clinical laboratories.

The sensitivity of the SAT-TB test in our study was 75.8% versus 89% for culture detection, which was somewhat higher than the 67.6% reported by Cui et al,^8^ but in their report the culture sensitivity was only 61.7%. In contrast, Fan et al^9^ reported sensitivities of 90% for bronchial washing fluid and 93% for sputum samples, with only slight differences to culture tests, and the sensitivity of the SAT-TB assay in smear-negative patients in their study was much higher than that in the current study (93.0% versus 72.3%). There are considerable sensitivity differences of SAT-TB tests, but it should be noted that the culture sensitivities differed in all studies using the same culture test method (Bactec MGIT 960). Fan et al tried to explain the SAT-TB sensitivity limitation with peripheral localized lesions, which would also explain the different results of the culture detection method. On the contrary, for nucleic acid amplification methods the presence of inhibitors of enzymatic amplification, a low number of mycobacteria, and/or an unequal distribution in the test suspension, are proposed reasons for sensitivity restrictions,^15,16^ which might also be the case for the SAT-TB method.

Our research differs from previous work in terms of its sample size and design. The 3000-cohort size was much larger than that of previous studies (generally <400 patients), so the bulk of our data is likely to be more objective and accurate. In addition, on repeated tests of sputum smears, the SAT-TB assay was performed once without knowing the status of the smear results, which often occurs in clinical practice.

Twenty patients with NTM lung disease were identified as false-positives by sputum smears but were negative according to the SAT-TB assay results, which showed a 0.743 agreement with culture methods. The current findings suggest that the SAT-TB assay is not only more sensitive for detecting MTB in sputum than a sputum smear test but also might alert physicians to NTM lung diseases in patients with a positive smear and a negative SAT-TB assay result. As SAT-TB assay results can be accomplished within 120 minutes, when a sputum sample is both SAT-TB assay and smear positive, it is recommended that anti-TB treatment be started in clinically suspected PTB without waiting for culture results (6–8 weeks). If a sputum sample is SAT-TB assay positive and smear negative, anti-TB treatment is still strongly recommended regardless of the results of smear and culture tests. If a sputum sample is SAT-TB assay negative and smear positive, tuberculosis could be a possible result of the diagnosis and anti-TB drugs are recommended as the first treatment. Subsequently, when the sputum culture results identify the disease as NTM, the treatment should be changed to anti-NTM medications. Conversely, cases in which both the SAT-TB assay and smear test results are negative, other pulmonary diseases should be considered first.

Our study is the first large cohort study (3000 patients) that has evaluated the SAT-TB assay in clinical practice. We found that the SAT-TB assay of sputum specimens could increase the TB diagnostic yield significantly, and is more useful than a sputum smear test. It is an accurate diagnostic method for PTB, especially in developing countries because of its low cost and rapidity. On the contrary, its low negative predictive value limits its usability to exclude the diagnosis of PTB and therefore SAT-TB-negative specimens still require conventional culture tests (solid or liquid) and specification. In addition, currently available technologies are not mutually exclusive.

As various PCR methods for the diagnosis of TB are introduced, verification and comparison will be necessary. Further multicenter studies are needed to confirm unequivocally the findings of the current study.
